# Potassium Overdose in Patient with Chronic Kidney Disease on Losartan: A Case Report

**DOI:** 10.5811/cpcem.47943

**Published:** 2025-12-07

**Authors:** Ahmed Naseem, Mark Schoenborn, James Scheidler, William Hunter Barclay, Garrett Volk

**Affiliations:** * McLaren Oakland Hospital, Department of Emergency Medicine, Pontiac, Michigan; † J.W. Ruby Memorial Hospital, Department of Emergency Medicine, Morgantown West Virginia; ‡ West Virginia University School of Medicine, Morgantown, West Virginia

**Keywords:** hyperkalemic emergency, chronic kidney disease, case report

## Abstract

**Introduction:**

Hyperkalemic emergencies can present with weakness, paralysis, sensorimotor deficits, and potentially fatal cardiac conduction abnormalities even in the absence of an elevated serum potassium. Common antihypertensive medications, such as angiotensin-converting enzyme inhibitors or angiotensin II receptor blockers, are associated with serum potassium elevations and can exacerbate hyperkalemia, especially in patients with renal impairment.

**Case Report:**

We report a 49-year-old patient who presented to the emergency department six hours following an intentional ingestion of potassium supplements totaling 600 milliequivalents (mEq). The patient also reported chronic use of ibuprofen and losartan 50 mg. Symptoms on presentation included weakness, chest pain, and shortness of breath. Initial labs revealed a potassium > 10 mEq/L which was beyond the upper limit of assay detection for metabolic testing. Calcium gluconate, insulin with dextrose, albuterol, sodium bicarbonate, calcium chloride, fluids, and furosemide were sequentially administered. Initial electrocardiogram (ECG) showed tachycardia, a widened QRS complex without discernible P waves, and non-specific ST-segment changes. Following treatment, a repeat ECG demonstrated decreased heart rate, normal axis, and a decreased QT interval. Creatinine at presentation was 1.67 mg per deciliter (patient’s baseline) with repeat labs revealing a potassium of 9.6 mEq/L. Definitive treatment with placement of a central venous catheter for emergent dialysis was initiated.

**Conclusion:**

This case illustrates how a patient’s regularly prescribed medication may complicate the management of an acute overdose. Prompt identification of a patient’s medications and supplements may expedite potentially life-saving interventions in a hyperkalemic emergency.

## INTRODUCTION

Hyperkalemic emergencies can be defined by various criteria related to elevated serum potassium and/or symptomatic presentation. Hyperkalemia is a common finding in patients with renal impairment and/or those who are prescribed renin-aldosterone-angiotensin-system inhibitors such as angiotensin-converting enzyme inhibitors (ACEi) or angiotensin II receptor blockers (ARB). While hyperkalemia is generally defined as a serum potassium greater than 5.0 milliequivalents per liter (mEq/L), a serum potassium greater than 6.5 mEq/L is often considered a hyperkalemic emergency. The presence of muscle weakness/paralysis or cardiac conduction abnormalities can also be considered a hyperkalemic emergency regardless of the severity of serum potassium elevation.

Neurologic and cardiac manifestations commonly occur in the setting of acute rises in serum potassium < 6.5 mEq/L.^1^ Intentional drug overdoses, such as with this patient, are a common presentation in the emergency department (ED). However, reports of attempted suicide with potassium supplements in patients with chronic kidney disease (CKD) are rare and even rarer in those concomitantly taking medications that can inhibit potassium excretion.

## CASE REPORT

A 49-year-old female with a history of hypertension, asthma, CKD stage III, stroke, and depression presented to the ED following a suicide attempt resulting in life-threatening hyperkalemia. Six hours prior to arrival, the patient intentionally consumed 30 pills containing 20 mEq of potassium. She also reported chronic use of nonprescribed ibuprofen and daily prescribed losartan 50 mg. The patient’s partner called emergency medical services after the onset of generalized weakness and confusion.

**Image 1:**
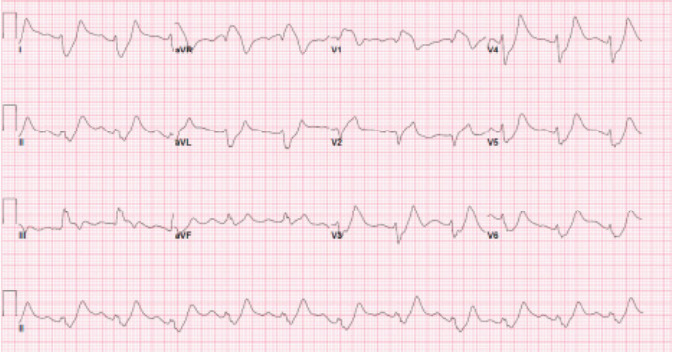
Pre-treatment electrocardiogram, prolonged QTc of 592 milliseconds, in a patient following intentional potassium overdose.

Upon arrival to the ED, the patient was weak but remained alert and oriented. She reported generalized weakness, chest pain, and shortness of breath. Vitals included the following: blood pressure, 131/83 millimeters of mercury; heart rate, 145 beats per minute (bpm); respiratory rate, 26 breaths per minute; and peripheral oxygen saturation, 100% on room air. Electrocardiogram (ECG) showed a heart rate of 68 bpm with right axis deviation, a significantly widened QRS complex without discernible P waves, and non-specific ST-segment changes (Image 1).

**Image 2:**
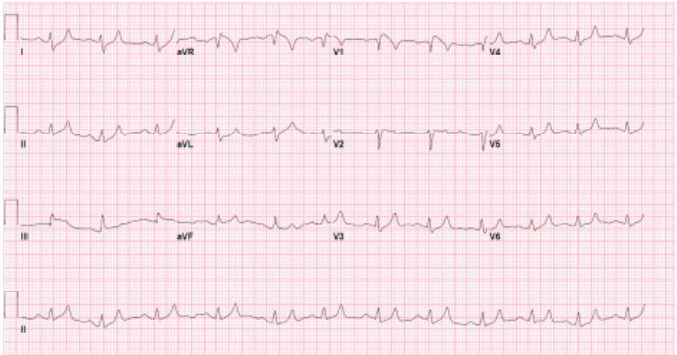
Post-treatment electrocardiogram, QTC decreased to 544 milliseconds, in a patient following management of her hyperkalemic emergency.

Serum potassium upon presentation was found to be greater than 10 mEq/L (reference range: 3.5–5.0 mEq/L) with an exact value beyond the upper limits of detection. The patient received intravenous (IV) calcium gluconate 2 grams (g), IV insulin 10 units, IV dextrose 50% 50 mL, nebulized albuterol 20 mg, IV sodium bicarbonate 100 mEq, IV calcium chloride 1g infusion, IV normal saline 1 L, and IV furosemide 40 mg. Shortly after treatment, repeat labs revealed a serum potassium of 9.6 mEq/L. Repeat ECG showed a heart rate of 104 bpm, and the QRS decreased from 128 to 112 milliseconds (Image 2). Blood glucose was 220 mg per deciliter (dL). Complete blood count, prothrombin time, international normalized ratio, blood gases, and troponin levels were within normal ranges. Nephrology was consulted, hemodialysis was initiated, and the patient was transferred to the intensive care unit with subsequent recovery and discharge.

*CPC-EM Capsule*

What do we already know about this clinical entity?

*Hyperkalemia management in emergency medicine is well-established, with progressive use of multiple agents providing timely, effective reduction of potassium levels.*

What makes this presentation of disease reportable?

*Our case highlights hyperkalemia management in a patient with renal impairment on a daily angiotensin II receptor blocker, causing rapidly rising potassium levels.*

What is the major learning point?

*Although standard therapies apply in an intentional potassium overdose with renal impairment, acuity rises due to rapid increases in potassium and subsequent poor excretion.*

How might this improve emergency medicine practice?

*Rapid recognition of hyperkalemia in renal impairment will aid emergency physicians in high-priority, complex cases.*

## DISCUSSION

Nearly half of ED visits related to hyperkalemia result in mortality. Patients with CKD and/or those taking an ACEi/ARB are at greater risk of developing hyperkalemia.^2^,^3^ Further, ACEi and ARB therapy have been associated with 10–38% of cases of hyperkalemia requiring hospitalization.^2^ These coexisting risk factors are of great importance in determining the prognosis of a hyperkalemic emergency given that taking higher doses of potassium does not always correlate with lethality. As illustrated by Simon, oral potassium doses of greater than 1,600 mEq/L can still be survivable while doses as low as 168 mEq/L may not.^4^

Management of hyperkalemia focuses on antagonizing the cardiac effects of potassium, shifting potassium into cells, and removing excess potassium from the body.^5^ Calcium, commonly administered as either calcium chloride or calcium gluconate, acts by directly antagonizing the cardiac membrane actions of potassium. Insulin’s role is to drive potassium into skeletal muscle cells via activation of the sodium potassium pump. If serum glucose is < 250 mg/dL, glucose should be administered to prevent hypoglycemia.^6^ Loop diuretics can also be added to promote potassium excretion via the urine. To then prevent further potassium absorption, oral potassium binding agents may be taken to promote excretion in the stool.^7^ Ultimately, hemodialysis may still be indicated for patients with acute or severe renal impairment.^8^

Although not used in this case because of proximity to rapid hemodialysis, gastrointestinal (GI) decontamination via both pharmacological and mechanical approaches remains a feasible way to limit continued potassium absorption in the setting of potassium ingestion. In cases of hyperkalemia resulting from enteral potassium exposure, decontamination represents one of the few treatment modalities capable of directly removing potassium from the body, whereas most standard therapies function by temporarily shifting potassium intracellularly.

The efficacy of GI decontamination is time sensitive and heavily dependent on the length of time from substance ingestion. Activated charcoal is often considered in the management of acute ingestion, but it is only effective in binding large, polarized organic compounds; generally it must be given within two hours of exposure, with the caveat of normal or near normal mental status to limit the risks of emesis and complications in airway management. Potassium is not bound by activated charcoal as it is a small, polarized, inorganic compound.

In contrast, whole bowel irrigation remains an option for many ingestions outside this short window. Whole bowel irrigation involves administering an osmotic laxative, such as polyethylene glycol, to help facilitate the expeditious removal of GI contents via the stool. However, while this method may be effective in reducing the total absorbable content of an acute ingestion, effectiveness is variable, and benefit in the literature remains debated. Importantly, this option would also be contraindicated in patients with hemodynamic instability or arrhythmia.^9^ Endoscopic removal has been described in the setting of a potassium salt bezoar. However, application of this technique is limited by the formulation of potassium ingested and the absence of clinically significant hyperkalemia in the report of this case.^5^

Long-acting, potassium binding agents remain an important adjunct for limiting the sustained absorption of extended formulation potassium supplements over a period of days.^10^ Examples more commonly used in practice include sodium polystyrene sulfonate and sodium zirconium cyclosilicate. Sodium polystyrene sulfonate is a non-specific cation-exchange resin that can be administered multiple times per day but carries a risk of GI complications, including colonic necrosis, and has questionable clinical efficacy.^11^ Sodium zirconium cyclosilicate, a selective potassium trapping agent, may be preferred as it is dosed daily and has a shorter onset of action.^10^

In this case, the use of sodium zirconium cyclosilicate was considered. However, its delayed onset of action—ranging from 1–4 hours post-ingestion—was deemed less favorable compared to immediate hemodialysis, which was readily available. Moreover, in the study by Amin et al, only 3.5% of patients had serum potassium > 6.5 millimoles per liter at baseline, and none had significant potassium ingestion as the etiology of their hyperkalemia.^12^ These differences limit the generalizability of those findings to our patient population. Despite these limitations, these pharmacologic agents may serve as a useful adjunct in cases where there is a delay in initiating dialysis or when managing ongoing enteral potassium absorption in conjunction with definitive therapy.

One of the unique features of this case report is that our patient presented with a baseline history of CKD and a creatinine of 1.67 mg/dL. Her chronic, nonsteroidal anti-inflammatory drug use, daily ARB, and intentional overdose all contributed to the hyperkalemic emergency. Corroborating history from family, along with supporting ECG changes, allowed for prompt initiation of potassium-lowering therapies. As reported by Madan et al, intentional potassium overdoses requiring hemodialysis occur infrequently. In this case series following 11 patients over an 11-year period, only five presented with an initial potassium > 7 mEq/L.^7^ In a case detailing a patient with concomitant pacemaker failure, Muck et al furthered the idea of this rarity by reporting that very few isolated case reports exist in the literature with an acute serum potassium > 10 mEq/L.^13^

## CONCLUSION

Cases of intentional potassium overdose must be managed swiftly and aggressively to prevent life-threatening outcomes. Even without the availability of exact laboratory values, patients’ existing comorbidities and medication history can quickly enable clinicians to classify them as high risk and allow for prompt disposition determination.
